# Ready-to-eat cereal consumption is associated with improved nutrient intakes and diet quality in Canadian adults and children across income levels

**DOI:** 10.3389/fnut.2023.1282252

**Published:** 2024-01-08

**Authors:** Lisa M. Sanders, Yong Zhu, Neha Jain, James Normington, Norton Holschuh, Megan Nechanicky, Michelle Tucker, Bibiana Garcia-Bailo

**Affiliations:** ^1^Cornerstone Nutrition, LLC, Battle Creek, MI, United States; ^2^Bell Institute of Health and Nutrition, General Mills, Golden Valley, MN, United States; ^3^Statistics and Data Science, General Mills, Mississauga, ON, Canada; ^4^Statistics and Data Science, General Mills, Golden Valley, MN, United States

**Keywords:** ready-to-eat cereal, nutrient intake, diet quality, socioeconomic status, Canadian Community Health Survey

## Abstract

**Background:**

Results from observational studies suggest ready-to-eat cereal (RTEC) consumption is associated with higher diet quality. In the United States, studies have shown that RTEC is an important contributor to nutrient intakes across income levels. However, it is unknown if this association varies by income level in the Canadian population. Given its affordability, RTEC may represent an important source of nutrients for lower-income individuals.

**Objective:**

This study evaluated the association of RTEC consumption with nutrient intakes and diet quality across household income levels in Canadian adults and children.

**Methods:**

Income and dietary data from 24 h dietary recalls were obtained from the 2015 Canadian Community Health Survey (CCHS)—Nutrition in 6,181 children (2–18 years) and 13,908 adults (19+ years). Diet quality was assessed with a modified Nutrient Rich Food Index (NRF) 9.3. Income levels were stratified into low, middle, and high based on family size, and data were analyzed by RTEC consumption and income level using multivariate linear regression adjusted for energy, age, and sex.

**Results:**

Diet quality was greater in adult and child RTEC consumers across all household income levels. Children and adults consuming RTEC also had higher nutrient intakes, including shortfall nutrients such as calcium, dietary fiber, iron, magnesium, and vitamin D. RTEC provided <10% of energy intake, <4% of saturated fat intake, and <9% of total sugar intake across all ages and income levels, while also providing one-third of daily iron intake and at least 10% of daily intake of dietary fiber, thiamin, folate, and vitamin B6.

**Conclusion:**

RTEC consumption was associated with improved nutrient intakes and diet quality in adults and children across household income levels. Nutrient dense and affordable food choices, such as RTEC, may be a helpful strategy to improve the diet quality of Canadians, particularly those with a lower household income.

## 1 Introduction

Poor diet quality has been identified as a key factor in the development of certain adverse health outcomes, such as type 2 diabetes, cardiovascular disease, and cancer (1, 2). Many of these health outcomes disparately impact individuals living in poverty or low-income conditions (3). Individuals living in low-income or food insecure households often have difficulty meeting nutrient needs, resulting in poor diet quality (4, 5). Thus, improving access to affordable, nutrient dense foods may improve diet quality in these populations and help minimize health disparities.

Ready-to-eat cereal (RTEC) is a convenient and affordable food that has been associated with improved diet quality and nutrient intakes in adults and children, and it can contribute to whole grain intake and encourage milk consumption (6–10). RTEC has also been associated with higher diet quality without increasing daily meal costs (11). With 90% of Canadians consuming breakfast on any given day (12), and RTEC being a food commonly consumed at breakfast, integrating RTEC into the diet may be a simple strategy to affordably improve diet quality.

A study in the US reported improved nutrient intakes and overall diet quality in children and adults consuming RTEC across all income levels (8), but it is not known if a similar relationship exists in other countries, such as Canada. The objective of this study was to investigate the contribution of RTEC to overall diet quality and specific nutrient intakes across income levels in a nationally representative sample of Canadian adults and children. Due to its affordability, RTEC may be an important contributor to nutrient intake and diet quality in lower income households.

## 2 Materials and methods

### 2.1 Data set and population

Data were obtained from the Canadian Community Health Survey (CCHS) 2015—Nutrition, a cross-sectional, nationally representative survey of individuals ≥ 1 year of age living in the 10 Canadian provinces (excluding indigenous populations on reserves, military personnel, and the institutionalized population). Data collected included two 24 h dietary recalls, measured height and weight, and a general health questionnaire with sociodemographic information (including income). According to Statistics Canada, household income was imputed if missing (13). A total of 20,483 participants completed Day 1 24 h recalls, which was a 62% response rate, and represented approximately 98% of the Canadian population. More information on the survey methodology and data collection is reported elsewhere (14). Dietary assessment via 24 h recalls was conducted using the automated multiple-pass method with trained interviewers. As previously described, this method employs 5 steps, including the time, amount, type, and location of food consumed in the previous 24 h. To minimize error and misreporting, prompts alerted the interviewer to confirm unusually high reported intakes of certain foods (13). Intake data for children 1–6 years of age were obtained via proxy, and children 6–11 years were assisted by a parent or guardian. Individuals 12 years and older completed the recall on their own with the interviewer. As only a subset of the CCHS 2015 participants completed a second day of 24 h recall, only data from Day 1 of the 24 h recalls were used, similar to previous studies (12, 15, 16).

In CCHS 2015—Nutrition, a total of 20,483 participants provided Day 1, 24 h dietary recalls. After excluding 372 infants and toddlers younger than 2 years and 22 subjects with missing income information, the present study included 6,181 children 2–18 years of age and 13,908 adults 19 years and older. The data evaluated in this study were de-identified and publicly available, and therefore did not require ethical review.

### 2.2 RTEC intake and income level

RTEC consumers were defined as anyone reporting RTEC consumption at any time on Day 1 of the 24 h recall. Self-reported income levels were stratified into low-, middle- and high-income groups based on the number of individuals in the household and the observed distribution of reported income in the population (Supplementary Table 1). The Government of Canada does not officially define poverty (17); therefore, the approach to income stratification based on household income distribution used in this study was chosen to provide adequate sample size across categories of income for statistical analysis.

### 2.3 Nutrient intakes and diet quality

Energy intake and intakes of 25 nutrients/food components were evaluated. The Canadian Nutrient File (18) was used to calculate nutrient intakes. The contribution of RTEC to the daily intake of nutrients was also assessed. The provision of whole grains from RTEC was provided in the CCHS-2015 Nutrition data set and assessed according to Health Canada’s 2014 Surveillance Tool (19). This tool categorizes whole grain foods into 4 tiers based on the degree of alignment with the 2007 Eating Well with Canada’s Food Guide (CFG). These tiers are determined by thresholds of intake for nutrients of public health concern (total fat, saturated fat, sodium, and total sugars) (19). Foods in tiers 1 and 2 are in line with CFG and contain little fat, total sugars, or sodium. Foods in tier 3 are partially aligned with CFG because they are high in either fat, total sugars, or sodium. Foods from tier 4 are considered not in line with CFG because they are typically high in two or more of fat, total sugars, and sodium (19).

Diet quality was assessed using a modification of the Nutrient Rich Food Index (NRF) 9.3, as previously described (12, 16). The NRF 9.3 compares the nutrition quality of food standardized to 2,000 kcal and has been validated against other established diet quality measures, including the Healthy Eating Index (HEI) (20). Briefly, the index is calculated by taking the sum of the percent daily value (DV) of nine nutrients to encourage (protein, fiber, vitamin A, vitamin C, vitamin E, calcium, iron, magnesium, and potassium) and subtracting the sum of the percent DV for saturated fat, added sugar and sodium. As described previously (12, 16), the modifications to the calculation in this study include replacing vitamin E with vitamin D and replacing added sugar with total sugar, because the Canadian Nutrient File does not contain data on added sugars. The updated Canadian DVs were used to calculate percent DV (21). For nutrients to encourage, intake at or above the percent DV was truncated at 100 and for nutrients to limit, intake below the percent DV was truncated at 0. Thus, the maximum score achievable was 900, representing intake at or exceeding the percent DV for all nutrients to encourage and not exceeding the percent DV for any nutrients to limit.

### 2.4 Statistical analysis

Statistical analysis was conducted with SAS (Version 9.4, SAS Institute), using Statistics Canada-recommended weighting procedures. Values are shown as means +/− standard errors (SE) and percentages, where applicable. Chi-squared tests were performed to compare the distribution of categorical demographic variables between RTEC consumers and non-consumers. The Student’s t-test was used to compare continuous variables. Analysis of covariance (ANCOVA) was used to evaluate differences across age and income groups in mean daily nutrient and energy intakes. Age, sex, and energy intake were used as covariates for nutrient intake and age and sex were covariates for energy intake. ANCOVA was also used to compare diet quality (via the NRF 9.3) across RTEC intake categories with only age and sex as covariates because NRF 9.3 is standardized to energy intake. Alpha was set at 0.05.

## 3 Results

### 3.1 Demographics of RTEC consumption

The characteristics of RTEC consumers and non-consumers by income level are included in Table 1. More than one-third of children were RTEC consumers, while less than 25% of adults reported consuming RTEC. The portion of children and adults consuming RTEC was similar across all income groups. Among children, RTEC consumers across all income levels were younger than non-consumers (*p* < 0.04). Alternatively, among adults, RTEC consumers were older than non-consumers (*p* < 0.05) across all income levels. There were no differences in RTEC intake by sex and income level.

**Table 1:** Demographic characteristics of Canadian children and adults by income and RTEC consumption, CCHS 2015—Nutrition.

Characteristics	Children and Adolescents 2–18 years (*n* = 6,181)			Adults 19+ years (*n* = 13,908)		
	RTEC consumers	RTEC non-consumers	Value of *p*	RTEC consumers	RTEC non-consumers	Value of *p*
***N* (% of population)**						
Low income	582 (36%)	1,043 (64%)		756 (22%)	2,701 (78%)	
Middle income	916 (35%)	1,696 (65%)		1,603 (23%)	5,400 (77%)	
High income	709 (36%)	1,235 (64%)		719 (21%)	2,729 (79%)	
**Age (mean ± SE, years)**						
Low income	8.6 ± 0.4	10.6 ± 0.2	**<0.0001**	56.3 ± 1.6	50.3 ± 0.8	**0.0005**
Middle income	9.3 ± 0.3	10.1 ± 0.2	**0.0359**	52.5 ± 1.0	49.5 ± 0.5	**0.0081**
High income	9.2 ± 0.3	10.6 ± 0.3	**0.0011**	47.5 ± 1.1	45.1 ± 0.6	**0.0478**
**Sex (% male)**						
Low income	48.7	51.2	0.5994	44.9	44.3	0.8985
Middle income	51.8	50.9	0.8152	46.9	47.9	0.7337
High income	50.2	47.7	0.5295	50.5	55.6	0.2279

*p*-values in bold represent significant differences between RTEC consumers and non-consumers; RTEC, ready-to-eat cereal.

### 3.2 Daily energy intake, nutrient intakes, and diet quality by income and RTEC consumption

Energy intake in children did not differ based on RTEC consumption, regardless of income level (Table 2). However, adult RTEC consumers in the low- and middle-income groups consumed significantly more energy than RTEC non-consumers (*p* < 0.023; Table 3). Daily energy intake did not differ among high-income adults based on RTEC consumption.

**Table 2:** Adjusted daily energy and nutrient intake in Canadian children by income and RTEC consumption, CCHS 2015—Nutrition.

	Low income (*n* = 1,625)			Middle income (*n* = 2,612)			High income (*n* = 1,934)		
	RTEC consumers	RTEC non-consumers	Value of *p*	RTEC consumers	RTEC non-consumers	Value of *p*	RTEC consumers	RTEC non-consumers	Value of *p*
**Energy and macronutrients**									
Energy (kcal)	1853.8 ± 44.8	1796.8 ± 38.4	0.3332	1880.6 ± 54.7	1809.4 ± 32.6	0.2524	1925.2 ± 51.0	1892.0 ± 36.1	0.5965
Carbohydrates (g)	254.8 ± 3.0	244.1 ± 2.5	**0.0043**	252.7 ± 3.0	243.1 ± 2.2	**0.0086**	255.0 ± 2.8	242.9 ± 2.1	**0.0007**
Total sugars (g)	112 ± 2.7	103.4 ± 2.3	**0.0127**	112.3 ± 2.7	107.0 ± 2.0	0.0993	116.1 ± 2.4	108.1 ± 1.9	**0.0101**
Dietary fiber (g)	16.2 ± 0.5	14.5 ± 0.3	**0.0020**	16.5 ± 0.4	14.4 ± 0.3	**<0.0001**	16.6 ± 0.4	15.6 ± 0.3	**0.0417**
Protein (g)	69.7 ± 1.4	67.8 ± 1.4	0.3195	74.3 ± 2.3	73.4 ± 1.1	0.7199	74.3 ± 1.9	73.4 ± 1.0	0.6772
Fat (g)	60.1 ± 1.0	65.5 ± 0.9	**<0.0001**	62.8 ± 0.9	67.0 ± 0.8	**0.0003**	64.2 ± 1.0	69.5 ± 0.8	**<0.0001**
Monounsaturated fat (g)	21.8 ± 0.5	23.4 ± 0.5	**0.0096**	22.1 ± 0.4	24.3 ± 0.4	**0.0002**	23.0 ± 0.5	25.0 ± 0.4	**0.0032**
Polyunsaturated fat (g)	11.3 ± 0.3	13.2 ± 0.4	**0.0009**	12.1 ± 0.3	12.9 ± 0.2	**0.0403**	12.5 ± 0.3	13.4 ± 0.3	**0.0319**
Saturated fat (g)	21.5 ± 0.6	22.9 ± 0.5	0.0728	22.9 ± 0.5	23.7 ± 0.3	0.1481	23.0 ± 0.4	24.8 ± 0.3	**0.0012**
Cholesterol (mg)	194.7 ± 8.7	222.9 ± 7.8	**0.0138**	208 ± 10.8	240.4 ± 6.9	**0.0102**	195.7 ± 8.3	244.5 ± 7.6	**<0.0001**
**Vitamins and minerals**									
Calcium (mg)	979.3 ± 34.9	827.9 ± 20.3	**0.0002**	1063.7 ± 22.3	894.7 ± 17.3	**<0.0001**	1072.9 ± 23.7	970.3 ± 17.3	**0.0005**
Folate DFE (μg)	424.6 ± 13.0	428.8 ± 11.4	0.8105	473.5 ± 15.8	430.7 ± 9.0	**0.0171**	445.3 ± 10.0	431.6 ± 9.2	0.3279
Iron (mg)	13.8 ± 0.3	11.0 ± 0.2	**<0.0001**	14.8 ± 0.3	11.0 ± 0.1	**<0.0001**	14.5 ± 0.4	11.1 ± 0.2	**<0.0001**
Magnesium (mg)	260.6 ± 6.0	245.7 ± 4.0	**0.0378**	286.0 ± 8.0	253.8 ± 3.6	**0.0002**	273.6 ± 5.2	260.0 ± 3.5	**0.0380**
Niacin (mg)	33.5 ± 0.7	32.3 ± 0.6	0.1923	35.8 ± 1.3	34.3 ± 0.6	0.2658	35.2 ± 1.0	34.3 ± 0.6	0.4515
Phosphorous (mg)	1262.5 ± 30.2	1161.2 ± 17.3	**0.0034**	1357.1 ± 26.6	1250.0 ± 15.1	**0.0004**	1345.1 ± 22.4	1291.4 ± 16.6	0.0584
Potassium (mg)	2459.4 ± 49.4	2275.2 ± 36.1	**0.0029**	2531.5 ± 39.0	2376.5 ± 34.7	**0.0030**	2494.6 ± 46.9	2436.6 ± 31.1	0.3134
Riboflavin (mg)	1.9 ± 0.1	1.7 ± 0.0	**0.0182**	2.0 ± 0.0	1.8 ± 0.0	**<0.0001**	1.9 ± 0.0	1.9 ± 0.0	0.3605
Sodium (mg)	2540.3 ± 63.2	2549.7 ± 43.9	0.9020	2598.6 ± 41.1	2651.2 ± 35.7	0.3248	2537.5 ± 45.5	2624.4 ± 41.0	0.1552
Thiamin (mg)	1.8 ± 0.0	1.4 ± 0.0	**<0.0001**	1.9 ± 0.0	1.5 ± 0.0	**<0.0001**	1.8 ± 0.0	1.4 ± 0.0	**<0.0001**
Vitamin A RAE (μg)	633.7 ± 92.4	536.2 ± 18.4	0.3081	682.6 ± 37.4	594.5 ± 17.8	**0.0345**	642.5 ± 25.1	620.4 ± 14.8	0.4540
Vitamin B12 (μg)	4.6 ± 0.9	3.4 ± 0.2	0.2336	4.1 ± 0.1	3.8 ± 0.1	0.1089	4.0 ± 0.1	4.0 ± 0.2	0.9420
Vitamin B6 (mg)	1.5 ± 0.1	1.4 ± 0.0	**0.0138**	1.6 ± 0.1	1.4 ± 0.0	**0.0002**	1.6 ± 0.0	1.4 ± 0.1	**0.0350**
Vitamin C (mg)	111.8 ± 6.0	120.2 ± 5.8	0.3036	113.9 ± 5.5	117.9 ± 3.9	0.5516	109.9 ± 5.5	115.4 ± 4.4	0.4322
Vitamin D (D2 + D3) (μg)	5.8 ± 0.3	4.8 ± 0.2	**0.0059**	6.7 ± 0.3	5.1 ± 0.2	**<0.0001**	6.5 ± 0.2	5.5 ± 0.2	**0.0036**
Zinc (mg)	9.3 ± 0.3	8.7 ± 0.3	0.1134	10.0 ± 0.3	9.5 ± 0.2	0.1084	9.8 ± 0.3	9.4 ± 0.2	0.2210

Adjusted for age, gender (energy intake), and total energy intake (nutrients only); *p*-values in bold represent significant differences between RTEC consumers and RTEC non-consumers within an income group; DFE, dietary folate equivalents; RAE, retinoic acid equivalents; RTEC, ready-to-eat cereal.

**Table 3:** Adjusted daily energy and nutrient intakes in Canadian adults by income and RTEC consumption, CCHS 2015—Nutrition.

	Low income (*n* = 1,625)			Middle income (*n* = 2,612)			High income (*n* = 1,934)		
	RTEC consumers	RTEC non-consumers	Value of *p*	RTEC consumers	RTEC non-consumers	Value of *p*	RTEC consumers	RTEC non-consumers	Value of *p*
**Energy and macronutrients**									
Energy (kcal)	1886.8 ± 47.9	1732.4 ± 31.3	**0.0071**	1943.0 ± 39.6	1840.5 ± 20.2	**0.0227**	1964.6 ± 44.1	1919.0 ± 31.9	0.4033
Carbohydrates (g)	229.4 ± 4.9	205.4 ± 2.0	**<0.0001**	237.0 ± 2.5	219.6 ± 1.5	**<0.0001**	242.1 ± 3.8	221.6 ± 2.2	**<0.0001**
Total sugars (g)	96.3 ± 4.3	77.5 ± 1.6	**0.0001**	96.4 ± 1.7	85.6 ± 1.2	**<0.0001**	95.9 ± 2.6	85.3 ± 1.7	**0.0007**
Dietary fiber (g)	18.9 ± 0.7	14.9 ± 0.3	**<0.0001**	19.9 ± 0.5	16.6 ± 0.2	**<0.0001**	21.3 ± 0.6	17.8 ± 0.3	**<0.0001**
Protein (g)	70.8 ± 1.5	70.8 ± 1.0	0.9928	78.6 ± 1.3	77.6 ± 0.7	0.4672	84.3 ± 1.5	85.1 ± 1.1	0.6667
Fat (g)	57.4 ± 2.0	63.7 ± 0.7	**0.0047**	66.1 ± 0.9	70.4 ± 0.6	**<0.0001**	70.5 ± 1.7	74.5 ± 0.8	**0.0345**
Monounsaturated fat (g)	20.3 ± 0.9	24.2 ± 0.4	**0.0001**	24.3 ± 0.4	26.4 ± 0.3	**0.0001**	25.8 ± 0.6	27.9 ± 0.4	**0.0024**
Polyunsaturated fat (g)	11.4 ± 0.5	13.2 ± 0.3	**0.0016**	13.6 ± 0.3	15.0 ± 0.2	**0.0002**	15.7 ± 1.0	16.0 ± 0.3	0.6938
Saturated fat (g)	20.3 ± 1.0	20.5 ± 0.3	0.8608	22.3 ± 0.4	22.9 ± 0.3	0.2255	23.1 ± 0.5	23.8 ± 0.4	0.2763
Cholesterol (mg)	201.5 ± 12	252.9 ± 9.0	**0.0009**	233.1 ± 10.1	273.9 ± 5.7	**0.0006**	229.1 ± 9.8	309.1 ± 10.9	**<0.0001**
**Vitamins and minerals**									
Calcium (mg)	851.4 ± 41.1	658.5 ± 14.7	**<0.0001**	896.2 ± 18.5	744.2 ± 10.2	**<0.0001**	963.1 ± 25.1	807.3 ± 15.8	**<0.0001**
Folate DFE (μg)	399.1 ± 14.2	396.2 ± 8.2	0.8656	446.4 ± 8.5	428.2 ± 4.7	0.0682	479.0 ± 13.1	471.4 ± 9.1	0.6326
Iron (mg)	14.1 ± 0.4	10.6 ± 0.2	**<0.0001**	15.0 ± 0.2	11.5 ± 0.1	**<0.0001**	15.7 ± 0.4	12.5 ± 0.2	**<0.0001**
Magnesium (mg)	303.4 ± 7.2	265.7 ± 3.4	**<0.0001**	331.8 ± 5.3	301.3 ± 3.2	**<0.0001**	352.4 ± 7.0	325.1 ± 4.7	**0.0012**
Niacin (mg)	34.9 ± 0.8	34.7 ± 0.5	0.8287	39.5 ± 0.6	38.3 ± 0.4	0.1305	42.2 ± 1.0	41.7 ± 0.6	0.6759
Phosphorous (mg)	1260.2 ± 25	1120.7 ± 14.2	**<0.0001**	1361.1 ± 18.3	1229.7 ± 9.1	**<0.0001**	1499.2 ± 35.2	1337.1 ± 17.8	**<0.0001**
Potassium (mg)	2612.9 ± 53.7	2383.8 ± 28.3	**0.0003**	2855.9 ± 41	2638.8 ± 22.4	**<0.0001**	3023.4 ± 62.2	2832.7 ± 33.9	**0.0073**
Riboflavin (mg)	1.9 ± 0.0	1.7 ± 0.0	**<0.0001**	2.0 ± 0.0	1.8 ± 0.0	**<0.0001**	2.2 ± 0.1	2 ± 0.0	**0.0295**
Sodium (mg)	2424.7 ± 85.5	2,534 ± 49.7	0.2748	2639.6 ± 41.3	2755.4 ± 27.1	**0.0216**	2653.5 ± 62.5	2853.7 ± 39.8	**0.0071**
Thiamin (mg)	1.9 ± 0.1	1.3 ± 0.0	**<0.0001**	1.9 ± 0.0	1.5 ± 0.0	**<0.0001**	2.1 ± 0.1	1.6 ± 0.0	**<0.0001**
Vitamin A RAE (μg)	575.3 ± 29.1	577.3 ± 27.0	0.9622	632.7 ± 20.5	653.2 ± 14.1	0.4156	718.7 ± 43.9	708.8 ± 24.5	0.8445
Vitamin B12 (μg)	4.0 ± 0.2	3.9 ± 0.2	0.6585	4.4 ± 0.3	3.7 ± 0.1	**0.0352**	4.6 ± 0.3	4.2 ± 0.1	0.2209
Vitamin B6 (mg)	1.7 ± 0.0	1.4 ± 0.0	**<0.0001**	1.8 ± 0.0	1.6 ± 0.0	**<0.0001**	2.0 ± 0.1	1.7 ± 0.0	**0.0002**
Vitamin C (mg)	96 ± 7.3	85.7 ± 4.0	0.2043	98.2 ± 4.4	97.3 ± 2.6	0.8608	103.0 ± 6.8	105.4 ± 4	0.7527
Vitamin D (D2 + D3) (μg)	5.6 ± 0.4	4.4 ± 0.2	**0.0060**	5.6 ± 0.3	4.5 ± 0.1	**0.0006**	5.8 ± 0.3	4.5 ± 0.2	**0.0009**
Zinc (mg)	10.1 ± 0.4	9.4 ± 0.2	0.1186	10.8 ± 0.3	10.1 ± 0.1	0.0503	11.7 ± 0.3	11.2 ± 0.2	0.1684

Adjusted for age, sex (energy intake only), and total energy intake (nutrients only); *p*-values in bold represent significant differences between RTEC consumers and RTEC non-consumers within an income group; DFE, dietary folate equivalents; RAE, retinoic acid equivalents; RTEC, ready-to-eat cereal.

Carbohydrate and dietary fiber intake were significantly higher in RTEC consumers compared to non-consumers across all ages and income levels (*p* < 0.042; Tables 2, 3). Total sugar intake was also significantly higher in RTEC consumers (*p* < 0.013), except middle-income children. Total fat and cholesterol intake were lower in RTEC consumers compared to non-consumers across all ages and income levels (*p* < 0.035). Children in the high-income group who consumed RTEC had a lower saturated fat intake compared to RTEC non-consumers (*p* = 0.0012), but saturated fat intake did not differ between RTEC consumers and non-consumers in other age and income groups. Protein intake was similar for RTEC consumers and non-consumers across all ages and income levels.

**Figure 1:**
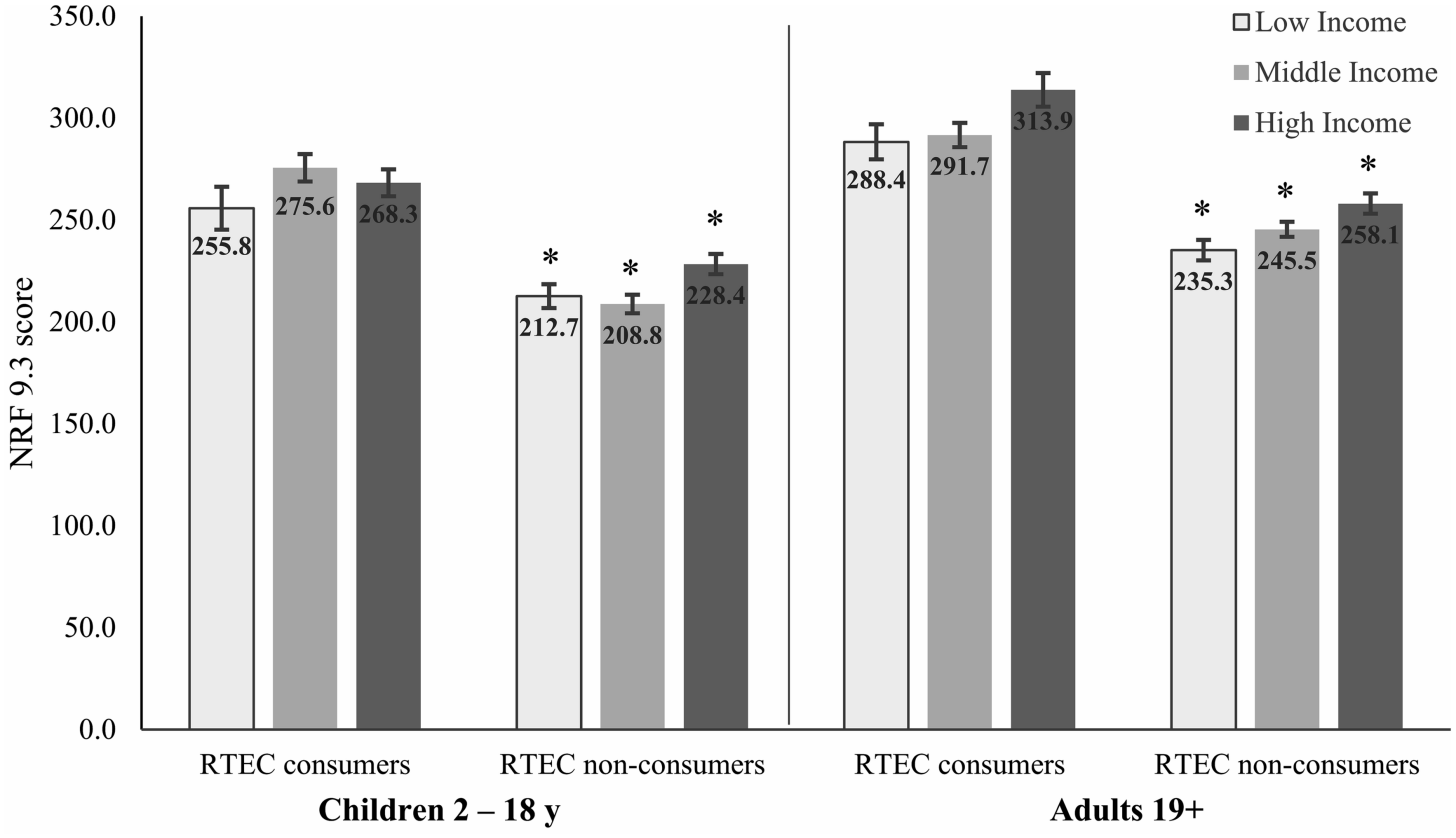
Adjusted diet quality (NRF 9.3) in Canadian children and adults by income and RTEC consumption. ^*^Significantly different from RTEC consumers in the similar age group and income level (*p* ≤ 0.0003). Low-, middle-, and high-income levels based on household size and reported in Supplementary Table 1. RTEC, ready-to-eat cereal.

Intake of often under-consumed nutrients, including calcium, iron, magnesium, potassium, and vitamin D, was greater among RTEC consumers across all ages and income levels (*p* < 0.038), with the exception of potassium intake in high-income children, which was similar between RTEC consumers and non-consumers. Additionally, intakes of thiamin and vitamin B6 were also greater among RTEC consumers across all ages and income levels (*p* ≤ 0.035). Folate and vitamin A intakes were only greater in child RTEC consumers at the middle-income level (*p* < 0.035). Sodium intakes did not differ in children based on income or RTEC consumption. However, middle- and high-income adults consuming RTEC had lower sodium intake than non-consumers (*p* < 0.022). A similar, non-significant trend was observed in low-income adults. Using the NRF 9.3, RTEC consumers of all ages and income levels had higher diet quality than non-consumers (*p* ≤ 0.0003; Figure 1).

### 3.3 Contribution of RTEC to nutrient intakes

**Figure 2:**
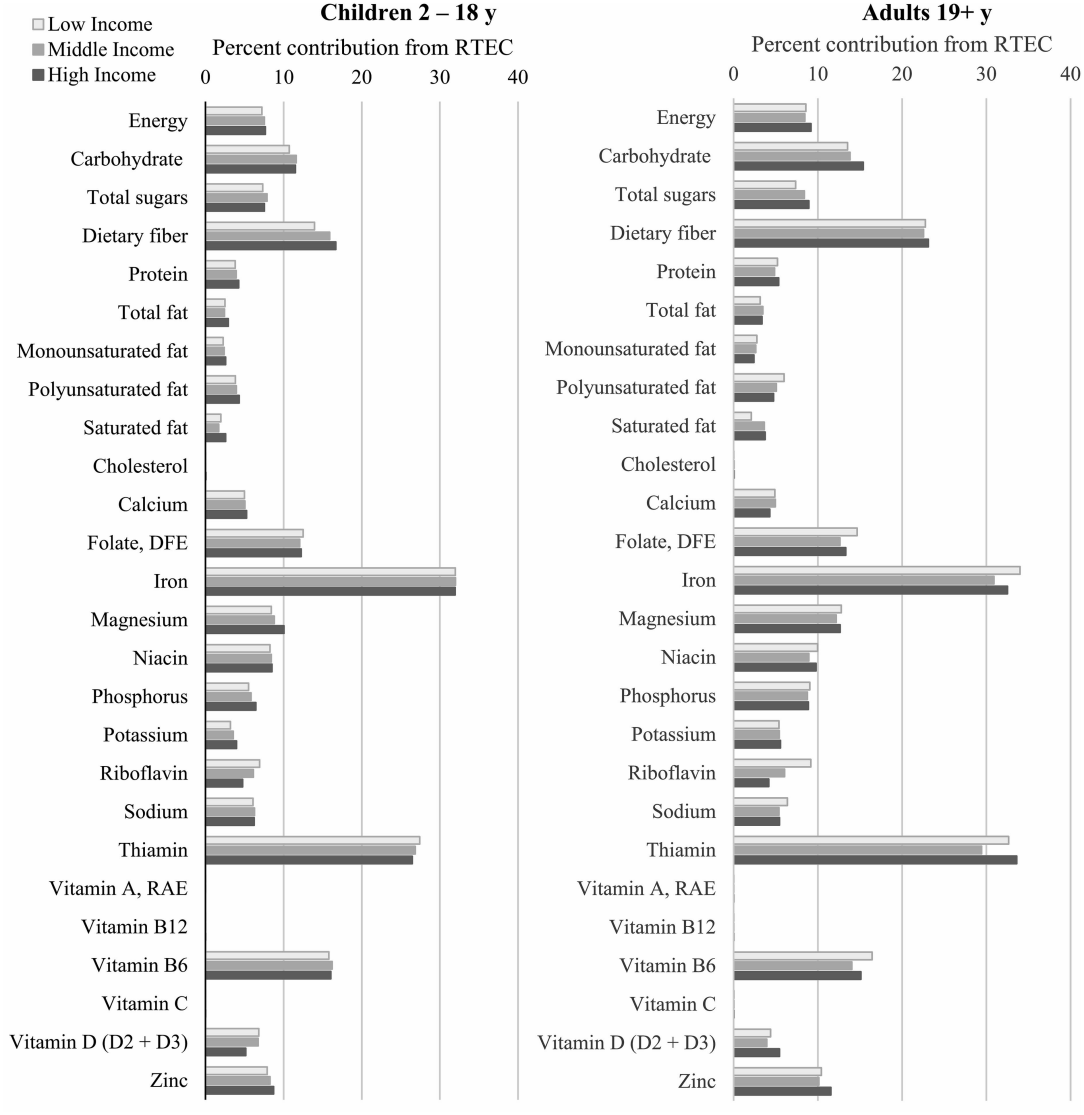
Percent contribution of RTEC to nutrient intakes of Canadian children and adults. RTEC, ready-to-eat cereal.

**Figure 3:**
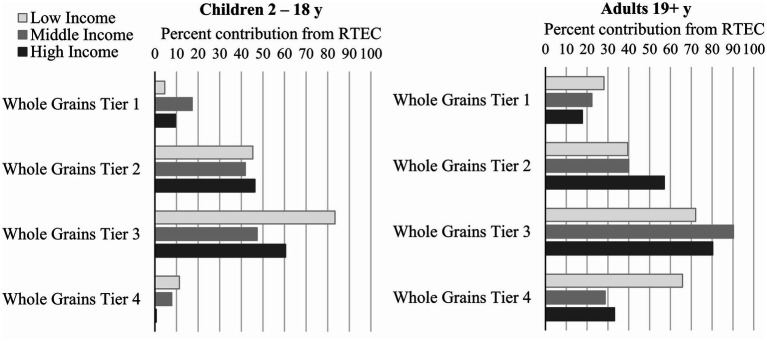
Percent contribution of RTEC to whole grain tiers in Canadian children and adults. RTEC, ready-to-eat cereal.

The percent contribution of RTEC to overall nutrient intakes and whole grain tiers is shown in Figures 2, 3. RTEC provided <10% of daily energy across all ages and income levels. RTEC provided one third of daily iron intake for all ages and income levels and at least 10% of daily intake of thiamin, folate, and vitamin B6. In children, RTEC provided more than 10% of daily dietary fiber, and in adults it provided more than 20% of daily dietary fiber intake. In adults only, RTEC provided at least 10% of magnesium and zinc. RTEC contributed <4% of saturated fat intake, < 9% of total sugar intake, and 5%–6% of daily sodium intake in adults and children across all income levels. RTEC did not contribute substantially to cholesterol, vitamin A, vitamin B12, or vitamin C intakes.

In children, RTEC contributed 47% to 83% of whole grains in tier 3 (partially aligned with CFG) and this was highest in low-income children (Figure 3). RTEC contributed approximately 50%–60% of whole grains in tier 1 and 2 (aligned with CFG) and <12% in tier 4 (not aligned with CFG). In adults, the percent contribution of RTEC was greatest for tier 3 whole grains, although the percentage of tier 1 and tier 2 were higher than that in children. Among adults, there was little difference in income levels, with the exception of tier 4 whole grains where the contribution from RTEC in low income consumers was approximately double that of middle and high income.

## 4 Discussion

The results presented here suggest that RTEC consumption is associated with higher diet quality and nutrient intakes, particularly shortfall nutrients such as calcium, iron, magnesium, potassium, and vitamin D, in Canadian children and adults across household income levels. RTEC also provided more than one-third of daily iron and whole grains and more than 10% of dietary fiber, thiamin, folate, and vitamin B6 across all ages and income levels. Additionally, RTEC was not a substantial source of nutrients to limit, contributing <4% of daily saturated fat, <9% of daily total sugars and 5%–6% of daily sodium. These observational findings suggest RTEC may positively contribute to nutrient intakes and diet quality, which may be particularly important in lower income households as a way to help offset disparities in diet-related health conditions.

The proportion of the population reporting RTEC consumption was similar across income levels in both children and adults, which may also partially explain why nutrient intakes and diet quality were similarly higher in RTEC consumers across all income levels. More than 35% of children and more than 20% of adults reported consuming RTEC, which is similar to previously reported results in the Canadian population (6), as well as the US population (~35% of US children and ~17% of US adults consume RTEC on a given day) (8). In Canada, RTEC remains a popular choice for children and adults and its intake does not appear to be influenced by income level. The mean age of child RTEC consumers tends to be younger, while the mean age of adult consumers tends to be older compared to RTEC non-consumers, in agreement with previous studies (6, 8) and similar to reported patterns of breakfast consumption in Canada (12).

The higher diet quality and nutrient intakes observed in adult and child RTEC consumers may be due to RTEC directly or to dietary patterns and specific foods that are associated with RTEC intake. RTEC, as a grain-based food, can be an important source of dietary fiber (22, 23) and, in Canada, is frequently fortified with micronutrients, such as niacin, folate, thiamin, vitamin B6, iron, magnesium, and zinc (6, 24). Daily intakes of dietary fiber and several of these micronutrients were significantly greater in child and adult RTEC consumers compared to non-consumers, and RTEC contributed 10% or more to the daily intakes of dietary fiber, folate, iron, thiamin, and vitamin B6 (as well as magnesium and zinc in adults only). Intakes of micronutrients such as calcium and vitamin D were also greater in RTEC consumers, which may be primarily from the milk that frequently accompanies RTEC consumption. While not evaluated in the current study, fruit consumption also frequently accompanies RTEC consumption and may further contribute to the observed differences in intakes of micronutrients (6, 8).

RTEC also contributed substantially to daily whole grain intake in children and adults. RTEC is one of the top sources of whole grain in the Canadian diet (16) similar to diets in several other countries (25–27). Yet, particularly among low-income adults, RTEC contributed a higher percentage of Tier 4 whole grains not aligned with CFG (19). Nevertheless, diet quality remained higher overall in low-income adults consuming RTEC compared to non-consumers, underscoring the importance of RTEC as a source of under-consumed nutrients, particularly for those living in low-income households.

Regarding nutrients to limit, such as sugar, sodium and saturated fat, RTEC consumers had similar or lower sodium and saturated fat intakes than non-consumers. However, total sugar intake was higher in RTEC consumers across all ages and incomes, with the exception of children in the middle-income group, who had a non-significant tendency to higher total sugar intake (*p* = 0.10). Barr et al. (15) also reported higher total sugar intake in Canadian children and adolescents consuming RTEC breakfasts; however, Vatanparast et al. (6) reported higher total sugar intake in adolescents and adults consuming RTEC, but not children. The differences in these findings may be due to the grouping of children and adolescents, or stratification by income level, as in the current study. Adults, regardless of RTEC consumption, consume, on average, less than the Daily Value (DV) for total sugars (100 g) established by Health Canada. However, children, regardless of RTEC consumption, consume more than the DV for total sugars. Overall, RTEC contributed <9% of total sugars across all ages and income levels. As the Canadian Nutrient File does not include information on added sugars, we were unable to ascertain what portion of the sugar consumed by CCHS 2015—Nutrition participants may come from sources such as milk and fruit, that frequently accompany RTEC consumption.

While higher diet quality for RTEC consumers compared to non-consumers was observed across all income levels, this may be particularly important for low-income consumers who could face challenges meeting nutrient needs (4, 5). A US-based study reported that intake of certain nutrients, such as dietary fiber and vitamin D, was positively associated with income (8). While not explicitly analyzed, a similar trend for dietary fiber and vitamin D can be observed in the current study. Adults and children living in low-income households, specifically those not consuming RTEC, have the lowest intake of these nutrients. However, intakes of vitamin D and dietary fiber in low-income RTEC consumers were similar to, or greater than, intakes observed in high-income RTEC non-consumers, emphasizing the potential role of RTEC in helping to overcome lower nutrient intakes that may occur at lower income levels. In the US, RTEC is frequently integrated into child and adult government feeding programs, and it is associated with adequate nutrient intake in at-risk and lower income populations (8). RTEC typically works well in these programs because of its affordability, acceptability, the variety of nutrients it offers, and its role in encouraging intake of other nutrient-rich foods, particularly milk. In Canada, despite limited government feeding programs, nonprofit programs typically integrate RTEC.

The strengths of this study include the use of a nationally representative data set with a validated approach to dietary assessment (multi-pass 24 h recall) and the adjustment for key confounders, such as age, sex, and energy intake. This is also the first study, to our knowledge, to assess the association of RTEC and nutrient intakes across income levels in Canada.

However, the study is limited by the cross-sectional nature of the data that prevents any inferences to causality. Additionally, the use of one 24 h recall may not represent normal consumption patterns or usual nutrient intake. Multiple 24 h recalls would provide a more robust estimate of usual intake. There also may be unaccounted-for residual confounding, particularly in adults, including health status, smoking, education, race/ethnicity, and rural/urban locations.

In sum, RTEC remains a frequently consumed food in the Canadian population and is associated with higher diet quality and nutrient intakes across all age and income levels. Particularly important for low-income Canadians, RTEC can be an affordable, nutrient dense food option to provide key nutrients and positively contribute to diet quality. Future dietary recommendations and nutritional policies should consider the potential role of affordable and nutrient dense foods, such as RTEC, in improving the diet quality of Canadian adults and children.

## Data availability statement

Publicly available datasets were analyzed in this study. This data can be found at: https://www150.statcan.gc.ca/n1/en/catalogue/82M0024X2018001.

## Author contributions

LS: Writing – original draft, Writing – review & editing. YZ: Conceptualization, Writing – review & editing. NJ: Formal analysis, Writing – review & editing. JN: Formal analysis, Writing – review & editing. NH: Formal analysis, Writing – review & editing. MN: Conceptualization, Writing – review & editing. MT: Writing – review & editing. BG-B: Supervision, Writing – review & editing.
