# Supplementary material for: Ready-to-eat cereal consumption is associated with improved nutrient intakes and diet quality in Canadian adults and children across income levels
Source: Front Nutr. 2024 Jan 8;10:1282252. doi: 10.3389/fnut.2023.1282252 (PMC10801256; doi:10.3389/fnut.2023.1282252)
Supplement: Supplementary file 1 [file Table_1.docx]

**Supplemental Data**

**Ready-to-eat Cereal Consumption is Associated with Improved Nutrient Intakes and Diet Quality in Canadian Adults and Children Across Income Levels**

**LM Sanders, et al.**

**Table S1.** Income levels (Canadian dollars) based on family size

| **Number of household members** | **Low income** | **Mid income** | **High income** |
| --- | --- | --- | --- |
| **1** | **≤19,999** | **20,000-79,999** | **80,000+** |
| **2 to 3** | **≤39,999** | **40,000-99,999** | **100,000+** |
| **4 to 5+** | **≤59,999** | **60,000-119,999** | **120,000+** |
